# The genetic aspects of multiple sclerosis

**DOI:** 10.4103/0972-2327.58272

**Published:** 2009

**Authors:** Stephen Sawcer

**Affiliations:** University of Cambridge, Department of Clinical Neurosciences, Addenbrooke's Hospital, Hills Road, Cambridge, CB2 2QQ, UK

**Keywords:** Genetics, genome-wide association study, multiple sclerosis

## Abstract

The epidemiology of multiple sclerosis has been extensively investigated and two features have consistently emerged: marked geographical variation in prevalence and substantial familial clustering. At first sight, geographic variation would seem to imply an environmental cause for the disease, while familial clustering would seem to suggest that genetic factors have the predominant etiological effect. However, given that geographic variation in prevalence could result from variation in the frequency of genetic risk alleles and that familial clustering might result from shared environmental exposure rather than shared genetic risk alleles, it is clear that these crude inferences are unreliable. Epidemiologists have been resourceful in their attempts to resolve this apparent conflict between “nurture and nature” and have employed a whole variety of sophisticated methods to try and untangle the etiology of multiple sclerosis. The body of evidence that has emerged from these efforts has formed the foundation for decades of research seeking to identify relevant genes and this is the obvious place to start any consideration of the genetics of multiple sclerosis.

## Epidemiology

**Figure 1:**
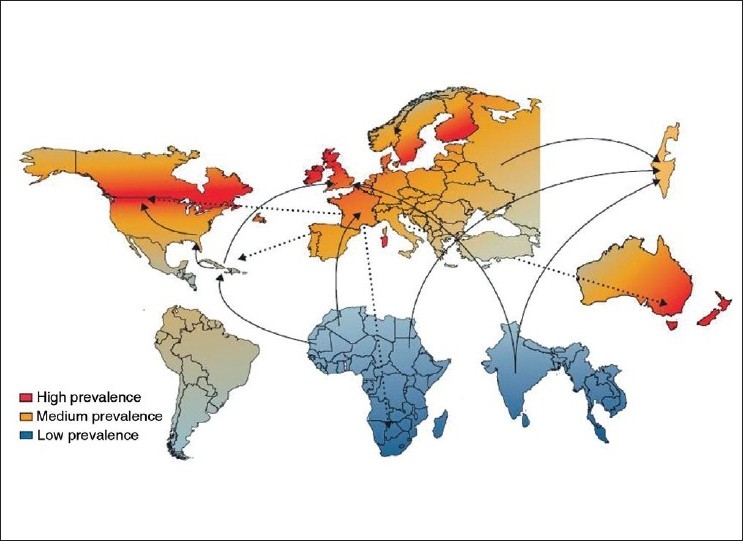
Global distribution of multiple sclerosis and migrations *The five continents are depicted, showing areas of medium prevalence of multiple sclerosis (orange), areas of exceptionally high frequency (red), and areas with low rates (grey-blue). Some regions are largely uncharted so these colors are only intended to provide an impression of the geographical trends. Major routes of migration studied from high-risk zone of northern Europe are shown as dotted arrows. Studies involving migrants from low-risk to high-risk zones are shown as solid arrows. Reprinted from The Lancet, Vol. 372, Compston and Coles, Multiple sclerosis, 1502-1517, Copyright (2008), with permission from Elsevier.[2]*

Although prevalence studies are difficult to perform and have an inherent tendency to underestimate the true frequency of disease, many such studies have been completed in multiple sclerosis and, together, they provide an almost global map of the distribution of the disease.[1] The core feature of this distribution is often summarized as a latitudinal gradient, with the disease being common in temperate regions and rare in topical climes [Figure 1].[2] Some authors have pointed out that the observed distribution also reflects the migration pattern of Northern Europeans, with the disease being common in those parts of the world in which Northern Europeans have settled.[3] In considering the global distribution of multiple sclerosis it is also important to note that there are exceptions to this general latitudinal gradient: some populations having rates of disease that are significantly different from that seen in their geographically nearby neighbors.[4] Studies looking at the risk of disease in migrant individuals moving from regions of low risk to regions of high risk, and vice versa[5–9] [Figure 1], suggest that risk changes with migration. These studies provide some of the clearest evidence supporting the role of environmental factors in the etiology of the disease. In any given part of the world, the risk of multiple sclerosis seems to be greatest in white individuals of European descent.

Amongst white individuals living in temperate regions such as Europe, Canada, and North America, 15–20% of those affected by multiple sclerosis report a family history of the disease—a rate which is significantly greater than would be expected given the prevalence of multiple sclerosis in these regions (typically 1 per 1,000).[1] Population-based studies of familial recurrence risk have confirmed and quantified the increased risk of the disease in the relatives of affected individuals.[10–12] This familial clustering can be usefully summarized as λ_s_ – the relative risk of the disease seen in the siblings of affected individuals as compared to the risk seen in the general population.[13] In multiple sclerosis this risk ratio takes a value of approximately 15[14] and, of course, reflects the combined effects of all shared etiological influences, both genetic and environmental.[15] In order to tease these influences apart, investigators have studied a variety of informative subgroups, including twins,[16–20] adoptees,[21] conjugal pairs,[22,23] half-siblings,[24] and step-siblings.[25] Taken together these data suggest that living with someone who has, or who will eventually develop, multiple sclerosis has little or no effect on one's risk of developing the disease unless one is genetically related to that person, in which case one's risk of having the disease increases with the degree of relatedness.[25] This is not to imply that environmental factors have no role, only that they seem to exert their effects mainly at a population level, with the micro-environmental differences between families within a given population seeming to be of relatively little importance.[25]

**Figure 2:**
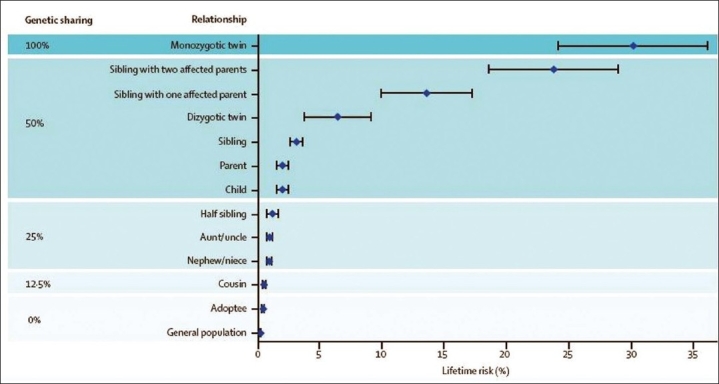
Recurrence risks for multiple sclerosis in families *Age-adjusted recurrence risks for different relatives of probands with multiple sclerosis and the degree of genetic sharing between relatives and the proband. Pooled data from population-based surveys. Error bars indicate 95% confidence intervals. Reprinted from The Lancet, Vol. 372, Compston and Coles, Multiple sclerosis, 1502-1517, Copyright (2008), with permission from Elsevier.[2]*

The epidemiology of multiple sclerosis continues to be studied, and interesting twists suggesting unsuspected possibilities will no doubt emerge in the future. However, as these studies are difficult to perform, are frequently subject to confounding and bias, and are also generally underpowered, it seems unlikely that they will ever provide any major insights. That said, it has been pointed out that the variation in recurrence risk with the degree of genetic relatedness can provide a useful indication of the genetic architecture underlying susceptibility to a complex disease.[26] In multiple sclerosis, these data [Figure 2] indicate that perhaps 100 common variants (those with a minor allele frequency of more than a few percent), each exerting only a modest effect on risk (increasing the odds of developing the disease by a factor of approximately 1.2–1.3) are likely to be involved.[27,28]

## Major Histocompatibility Complex

Early attempts to identify susceptibility genes in multiple sclerosis were highly successful and quickly identified the now well-established relevance of the Major Histocompatibility Complex (MHC) on chromosome 6p21. The first successes emerged in the early 1970s, when investigators showed that multiple sclerosis was associated with various Human Leukocyte Antigens (HLA), in particular HL-A3,[29] HL-A7,[30] and LD-7a[31] (in today's nomenclature HLA-A3, HLA-B7, and DR2, respectively). It was quickly realized that these associations were not independent but were, rather, a reflection of linkage dysequilibrium (LD; the tendency for certain alleles from linked loci to occur together on the same chromosome more often than you would expect by chance alone) between the corresponding alleles.[32,33] Over the years, technology has improved and the resolution of the associated alleles has been refined.[34–36]

The MHC is a gene-dense region of the genome that is characterized by extensive LD and extreme levels of polymorphism.[37] In light of these features, it is not surprising to find that many variants from this region show association with multiple sclerosis as a result of LD (correlation) between these various associated alleles.[38,39] The modest levels of LD between the class I region (containing the *HLA-A* and *HLA-B* genes) and the class II region (containing the *DRB1* and *DQB1* genes) enabled researchers to quickly establish that association primarily derived from the class II region.[32,33] However, the more extensive LD between *DRB1* and *DQB1* made it much more difficult to refine which of these genes was primarily responsible for the association. Studying African American patients, who have less intense LD between DRB1*1501 and DQB1*0602, Oksenberg *et al*.[40] provided the first convincing evidence that the primary association was with the *DRB1* gene, an observation which has been confirmed in subsequent studies in large cohorts of patients of European descent.[39]

Our understanding of the association between multiple sclerosis and the MHC continues to improve. It is now well established that MHC haplotypes other than just those carrying the DRB1*1501 allele also exert influence on susceptibility, either independently[41] or by modifying the risk associated with *1501.[42–44] What is unclear is whether or not these additional signals stem primarily from the *DRB1* gene or from the effects of other MHC loci. Many researchers have found evidence supporting the existence of an independent signal from the class I region,[39,41,45–48] while others have not.[38,49] Establishing the presence of additional susceptibility loci located close to a primary locus is a complex problem, especially in the presence of prominent LD and likely allelic heterogeneity.[50] The necessity to correct for the effects of LD with the DRB1*1501 allele limit the power of studies based on white European populations in which this allele is common.[38,39,42,43] Despite these difficulties, a role for the DRB1*03 haplotype is now well established[39,42,43] and Sardinian data would suggest that this association, at least, most likely stems from the *DRB1* gene.[41] The nature and origin of the remaining signals from the MHC region remain unresolved.

The role of HLA genes has also been studied in Asian multiple sclerosis. Serological studies performed in the 1980s found association with HLA-B12[51,52] rather than with B7, while molecular genetic analysis of the class II HLA genes, *DRB1*, *DQA1*, and *DQB1*, revealed the expected association with the European susceptibility haplotype DRB1*1501 −DQB1*0602.[53] In Asians, the DRB1*1502 and DRB1*16 subtypes of DR2 are more common than in Europeans and the extent of LD between DRB1*1501 and DQB1*0602 is significantly less intense.[54] In an analysis of Indian migrants living in the US, Rosenberg *et al*.[55] found that there was surprisingly little variation in ancestry across India, although there was a clear difference between Indian Asians and other ethnic groups.

By cataloguing variation in the MHC through the resequencing of specific haplotypes[56] and empirically establishing the complex patterns of LD across the region,[57] it has been possible to establish a comprehensive panel of haplotype-tagging Single-Nucleotide Polymorphisms (SNPs).[58] These SNPs are currently being typed in multiple sclerosis and a number of other autoimmune diseases as part of the International MHC and Autoimmunity Genetics Network (IMAGEN) project. Hopefully these systematic fine-mapping efforts will help to unravel this complex association, although it can be anticipated that large sample sizes will be needed to confirm the findings emerging from IMAGEN.

Other than the findings related to the MHC, the genetic analysis of multiple sclerosis has only very recently started to yield to the efforts of researchers. Prior to 2007, the lack of any convincing progress was a source of great frustration, and the inconsistency in early claims was rightly criticized.[59] It is now clear that two main issues have confounded the identification of relevant genes – the modest size of effects attributable to individual loci[60] and the failure to correctly allow for the statistical consequences that result from the enormous size of the genome.[61] The search for the genes of relevance in multiple sclerosis has been likened to searching for a handful of rather small needles in a very large haystack.[62,63]

### Linkage studies

Although available epidemiological data confirms that genetic factors are unequivocally relevant in multiple sclerosis, large extended families with multiple affected individuals in multiple generations are extremely uncommon. Most families contain no more than two or three affected individuals and no clear mode of inheritance can be inferred.[1] Parametric linkage analysis of the few larger families that have been described[64–68] has failed to identify any rare penetrant alleles that influence the risk of developing multiple sclerosis.

Since large extended families are not available for study, researchers have relied on nonparametric methods of linkage analysis based on looking for excess sharing of alleles amongst related individuals, most typically affected sibling pairs. In 1996, the results from the first attempts to systematically screen the genome for linkage to multiple sclerosis were reported in back-to-back publications from three populations – the UK,[69] the US,[70] and Canada.[71] Each of these studies was based on approximately 100 affected sib pairs and employed 300–400 microsatellite markers. Subsequently, similar studies were reported from Finland,[72] Sardinia,[73] Italy,[74] Scandinavia,[75] Australia,[76] and Turkey[77] and, in addition, each of the original three groups extended their analysis using further families and more microsatellite markers.[78–80] Disappointingly, none of these studies identified any statistically significant linkage, not even in the region of the MHC. Attempts at meta-analysis of these linkage data were no more successful, although linkage to the MHC just reached genome-wide significance in some of these studies.[81,82] In order to compensate for the inadequacies inherent in low-resolution microsatellite-based studies[83] the International Multiple Sclerosis Genetics Consortium (IMSGC) typed 4,506 SNPs in 730 multiplex families from Australia, Scandinavia, the US, and the UK. The enhanced power provided by this SNP–based screen is evident from the overwhelming evidence for linkage found in the MHC region, where a lod score of 11.7 was observed.[84] Once again, however, no other region of statistically significant linkage was identified. The comprehensive marker map used in this study makes it virtually impossible that any signals of a magnitude similar to that attributable to the MHC could have been missed. As with the previous studies, the number of suggestive linkage peaks was significantly greater than would have been expected to occur by chance alone,[84] indicating that there is excess allele sharing but providing no clear guide as to the location of the relevant genes.

Although these linkage data provide no useful information concerning the location of non-MHC susceptibility loci, the observed allele sharing does provide useful guidance concerning the size of effects attributable to such loci.[13] Employing the approach suggested by Risch and Merikangas,[85] and remembering that the observed allele sharing is expected to provide a significantly inflated estimate of effect size,[86] it is a straightforward matter to show that common non-MHC risk alleles are highly unlikely to increase the risk of the disease by more than a factor of 2. Under these circumstances it is clear that further linkage analysis is almost certain to be unrewarding since the number of sib pair families necessary to demonstrate significant linkage is impractically large.[85] These extensive linkage efforts are consistent with the findings from segregation analysis in suggesting that common risk alleles in multiple sclerosis are highly unlikely to exert more than a modest individual effect on risk. Fortunately, association-based studies are significantly more powerful and thus provide a means to identify genes exerting effects that fall below the resolution of linkage analysis.[85] However until recently studies looking at candidate genes in multiple sclerosis generally only considered modest numbers of cases, with the result that most of these studies were seriously underpowered to detect effects of the size now realized to be relevant. On the other hand this also means that there are no loci investigated prior to 2007, with the possible exception of APOE,[87] where published studies have been adequately powered to confidently exclude the possibility of a meaningful effect. It seems highly likely that many of the entirely plausible candidates considered to have been excluded on the basis of the absence of any consistent evidence to date will eventually emerge as genuinely relevant in the disease. It is surely the virtual absence of any power that is responsible for nearly all the apparent inconsistency in the literature concerning the genetics of multiple sclerosis.[88]

### Association studies

In the human population as a whole the total number of genetic variants runs into billions. However, most of these are exceedingly rare[89] and only around 10 million have a minor allele frequency (MAF) of greater than a few percent.[89,90] Although these common variants represent only a small fraction of the total number, they are responsible for 90% of the genetic difference between any two individuals.[26] Given that segregation analysis suggests that around 100 of these 10 million common variants might influence susceptibility to multiple sclerosis (see above) it is clear that the odds that any randomly chosen common variant is relevant in multiple sclerosis are approximately 100,000 to 1 against. These very low prior odds have a profound influence upon the odds that any nominally significant result identified in an association study is a true positive rather than false positive (the posterior odds).[91]

**Figure 3:**
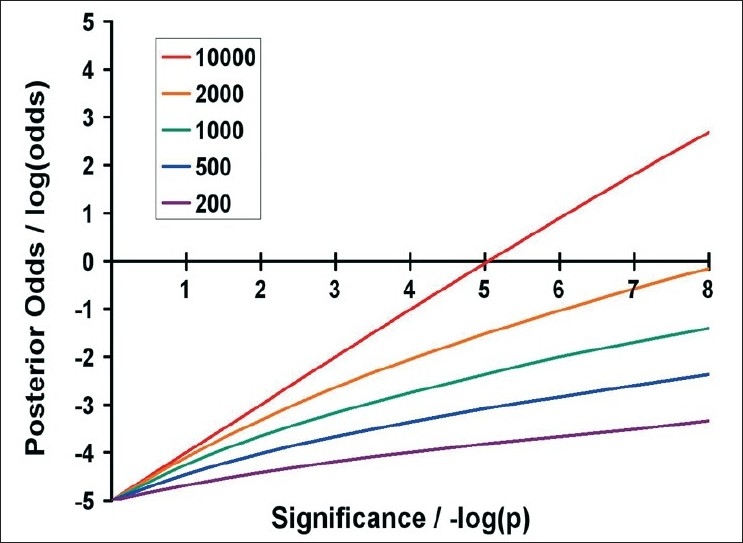
Posterior odds that a result is true, assuming risk alleles with a frequency of 10% and a genotype relative risk (GRR) of 1.2 and a multiplicative model *This figure indicates the posterior odds that a result is true (plotted on a log scale on the y-axis) against the significance of the result (plotted as the negative log of the *P*-value on the x-axis). Five sample sizes are listed in the legend; in each, the number of cases and controls are equal. The 200 line thus indicates the posterior odds for a study involving 200 cases and 200 controls, and so on. Power was calculated using the online genetic power calculator.[92] Reprinted from Brain, Vol. 131, 3118-31, Copyright (2008), with permission from Oxford University Press.[63]*

From Figure 3 it is obvious why *P*-values in the range of 5% to 0.1% will nearly always be false positives, even in well-powered studies. Importantly, Figure 3 also shows that the lower the power of a study, the more extreme the *P*-value must be before any result becomes more likely to be true rather than false.[93] The Wellcome Trust Case Control Consortium (WTCCC) proposed this Bayesian approach and through similar reasoning suggested that association studies in complex diseases should employ a minimum of 2,000 cases and that in this setting *P*-values of < 5 × 10^−7^ would more likely be true than false.[93]

One way to improve the likelihood of success in an association study is to use additional sources of information, such as animal models or expression data, to guide the selection of variants and thereby improve the prior odds. This candidate gene approach formed the bedrock of association studies in multiple sclerosis prior to the advent of Genome-Wide Association Studies (GWAS). However, even when multiple sources of alternate information are employed in a systematic way (so-called genomic convergence[94]) it is unlikely that the prior odds can be reduced below 1000:1.[91] In 2005, Fernald *et al*.[95] applied genomic convergence to multiple sclerosis and identified a short list of candidate genes, including the interleukin 7 receptor (*IL7R*), a locus that had previously been considered by Australian[96] and Swedish researchers.[97] In a follow-up study involving collaborators from the UK and Belgium, these US researchers studied 197 multiplex families, 1,901 trio families, 1,515 cases and 3,204 unrelated controls and identified a highly significant association (*P* = 2.9 × 10^−7^) with rs6897932 a non-synonymous SNP (nsSNP) from exon 6 of the *IL7R* gene,[98] a result which was replicated in an accompanying paper from Swedish researchers.[99] This locus was thus the first non-MHC susceptibility gene to be identified in multiple sclerosis.

**Figure 4:**
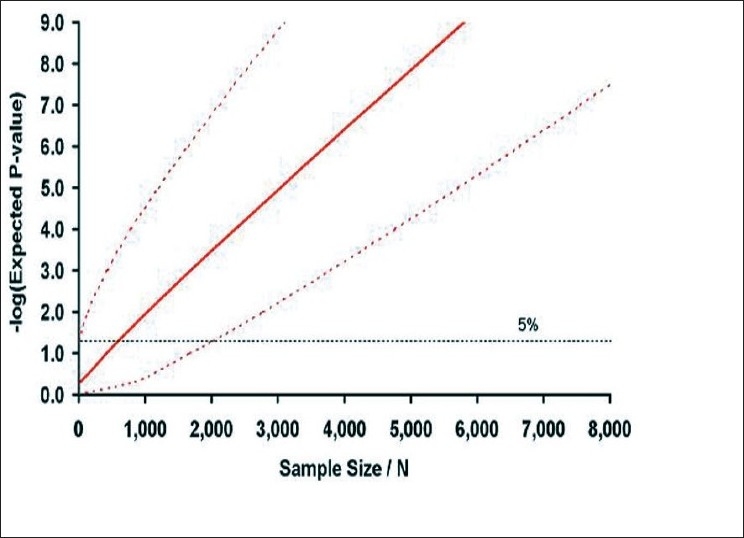
Expected *P*-value in follow-up studies of rs6897932, the IL7R-associated SNP *The red line indicates the expected *P*-value and the dotted lines the 95% confidence intervals on this estimate (plotted as the negative log). It can thus be expected that 95% of the time the observed *P*-value will fall within this space. The horizontal dotted line indicates the nominal 5% significance level. Reprinted from Brain, Vol. 131, 3118-31, Copyright (2008), with permission from Oxford University Press.[63]*

It is worth pausing to consider the nature of this association. The multiple sclerosis–associated allele of rs6897932 has a frequency of 72%, which means that approximately 9 out of every 10 white Europeans carry this risk allele. The allele is estimated to increase the risk of the disease by a factor of just 1.2. Using these parameters, we can calculate the *P*-value that would be expected in studies attempting to replicate this finding as a function of the sample size considered, as shown in Figure 4.

It is clear from Figure 4 that studies attempting to replicate association with effects of this size need to involve at least 2,000 cases and 2,000 controls if they are to have > 95% power to demonstrate nominally significant *P*-values (5%). Most attempts at replication involving more than 600 cases and 600 controls can be expected to yield a *P*-value of < 5% but not all. Studies with less than 600 cases and 600 controls are unlikely to identify even nominally significant association. It is important to keep these values in mind when interpreting replication studies. If a study involving just 400 cases and 400 controls fails to identify nominally significant association, this should not be interpreted as evidence that the association is not relevant in the tested population; in fact, this is perhaps the least likely explanation. Given the effect size and allele frequency, we can also calculate that the lod score that rs6897932 would be expected to generate in a set of 100 sib pairs is just 0.01. It is therefore clear that loci such as rs6897932 would not be expected to generate any linkage signals discernable in previously published linkage screens, and that any apparent concordance between identified susceptibility loci such as *IL7R* and previously reported linkage peaks is entirely coincidental.

Another way to improve the chances of identifying susceptibility genes would be to consider all common variation rather than just a single randomly selected or candidate variant. If all common variation were to be typed in a study, then this study would be sure to include an analysis of the relevant variants. In this situation, concerns about prior odds might be ignored and tests simply interpreted after some correction for multiple testing. However, rather predictably, nothing is gained by adopting this approach to analysis since the statistical penalty required to correct for multiple testing is equivalent to that incurred by allowing for the prior odds.[100] This is not surprising since both are simply a reflection of the size of the genome. This type of comprehensive (direct) GWAS would seem to be ideal, but in fact the extent of LD between common variants is so extensive that an indirect screen involving just a fraction of the markers relying on LD between tested and untested variants enables a large proportion (typically >80%) of common variation to be screened in a highly efficient manner.[101] Direct GWAS remains unaffordable and impractical at this time, whereas available technology means that indirect GWAS are possible and have proven to be a highly successful means of identifying the common variants influencing susceptibility to complex diseases.[90]

## GWAS in multiple sclerosis

In 2007, the IMSGC published the first GWAS completed in multiple sclerosis. In this study, 931 trio families (half from the US and half from the UK) were screened using the Affymetrix 500K chip, this yielded usable data from 334,923 SNPs.[102] The limited power provided by this modest number of trios meant that no unequivocal associations were identified in the screening phase, outside of the expected signals from the MHC. However, by utilizing additional control data from the WTCCC (*n* = 1,475) and the National Institutes of Mental Health (NIMH) (*n* = 956) along with candidate gene information, 110 SNPs were identified and followed up in an additional 609 trio families, 2,322 cases and 2,987 controls. In the final analysis (employing a total of 12,360 individuals), association with rs6897932 (from *IL7R*) was confirmed and significant association was also established with two SNPs from the interleukin-2 receptor (*IL2R*) gene, rs12722489 (*P* = 3.0 × 10^−8^), and rs2104286 (*P* = 2.2 × 10^−7^).[102] Studying these three SNPs in additional cohorts from Australia, Belgium, Denmark, Finland, Germany, Ireland, Italy, Netherlands, Norway, Sardinia, Spain, and Sweden, the IMSGC went on to extensively replicate these findings and were also able to show that the association with rs12722489 was entirely secondary to LD with rs2104286.[103]

Several investigators have observed an increased risk of a second autoimmune disease in the families of individuals affected by multiple sclerosis.[104,105] This familial clustering of autoimmune diseases immediately suggests the possibility that some genetic variants might influence the risk of autoimmunity in general and, therefore, that risk alleles for one autoimmune disease might make logical candidates for multiple sclerosis. Following this logic, IMSGC typed seven SNPs established as being relevant in type 1 diabetes in 2,369 trio families, 5,737 cases and 10,296 controls. This analysis confirmed highly significant association with rs12708716 from the C-type lectin domain family 16, member A (*CLEC16A*) gene (*P* = 1.6 × 10^−15^) and rs763361 from the *CD226* gene (*P* = 5.4 × 10^−8^).[106] Both these associations were replicated in accompanying papers.[107,108]

The CLEC16A gene (aka KIAA0350) had been identified as potentially associated in the original IMSGC GWAS,[102] as had CD58. Follow-up efforts in CD58 have also confirmed this association.[109]

In 2007, the WTCCC also produced a multiple sclerosis GWAS[110] that was based on 975 cases and 1,466 controls screened with 12,374 nsSNPs. Again, the limited power provided by the cohort size meant that the screen failed to identify any unequivocally associated markers. However, it is relevant to note that rs6897932 was the eighth most associated marker identified, further confirming that a GWAS–based approach would have identified this association had it not already been established through genomic convergence and the candidate gene approach.

The recent publication of the ‘Gene MSA’ (Multiple Sclerosis Association) GWAS,[111] the product of a collaboration between US, Dutch, and Swiss researchers (and supported by GlaxoSmithKline), brings the total of multiple sclerosis GWAS to three. This study typed the Illumina HumanHap550 chip in 978 cases and 883 controls and considered extensive phenotyping data, including that from magnetic resonance imaging (MRI). As would be expected, there were no unequivocal associations outside of the MHC, but association with the Glypican-5 (GPC5) locus was suggested, and further supported in an additional 974 US cases. Table 1 shows the current list of established non-MHC risk alleles for multiple sclerosis.

**Table 1:** Established multiple sclerosis non-MHC risk alleles

Implicated gene	Associated SNP	RAF/%	Odds ratio
IL7R	rs6897932	72	1.2
IL2R	rs2104286	72	1.3
CLEC16A	rs12708716	65	1.2
CD226	rs763361	47	1.1
CD58	rs2300747	89	1.3
GPC5	rs9523762	35	1.3

RAF = Risk Allele Frequency / %

### The future

Further GWAS in multiple sclerosis are expected to emerge in the near future and will no doubt add to the growing list of established candidate susceptibility genes. Refining these associations and understanding how these variants exert their effect will engage researchers for some time but seems likely to shed new light on our understanding of this enigmatic disease.[90]

Note added in proofs a further multiple sclerosis GWAS has been reported since this review was contructed - Australia and New Zealand Multiple Sclerosis Genetics Consortium (ANZgene). Genome-wide association study identifies new multiple sclerosis susceptibility loci on chromosomes 12 and 20. Nat Genet. 2009 41:824-8.
